# Practical Observations in Ophthalmology

**Published:** 1875-05

**Authors:** J. O. Stilson

**Affiliations:** Late Clinical Assistant to Dr. E. Williams, Cincinnati


### PRACTICAL OBSERVATIONS IN OPHTHALMOLOGY.

BY J. O. STILSON, A. B. M. D., LATE CLINICAL ASSISTANT TO DR. E. WILLIAMS, CINCINNATI.

*Read before the Mitchell District Medical Society, August 7th,* 1874.

Inflammation of the cornea—keratitis—may be either acute or subacute, and acute keratitis may be either diffused or circumscribed. Diffused corneitis is characterized by an opaque appearance, hazy or nebulous condition of the cornea either throughout its entire extent or in one or more places near each other. The parts most usually affected at the beginning of the attack, are near the margin, and afterwards the disease spreads, involving the more central portions of the cornea. The opaque appearance frequently presents the form of ground glass, caused by a roughening of the external epithelial layer of the cornea. The vessels of the conjunction become injected and tortuous, and sometimes will be seen to encroach upon the margin of the cornea and run toward the centre. In addition to the increased size of the conjunctineal vessels, the sclerotic ring or zone will be found to be highly injected, and on account of extreme minuteness of the vessels and their great number, the edge of the sclerotic all around the cornea will present a uniform pinkish appearance.

The amount of vascularity of the sclerotic will vary with the intensity of the attack. In subacute or chronic cases, or in cases due to paralysis of the fifth nerve, where the roughness and abrasion have been brought on by exposure to air, the cornea will be of a dull gray, or lifeless appearance, without much vascularity, but where the disease is acute, and especially where there are several small patches of ulceration throughout the substance of the cornea, the ciliary injection will be quite intense and the vascularity increased.

The subjective symptoms are pain, photophobia, dimness of vision and lachrymation.

These subjective symptoms will vary considerably according to the attack and the extent to which the structures are involved. In simple inflammation of the cornea where the epithelial layer is not abraded or ulcerated, and where the minute nerves remain unexposed, or in subacute ulceration, where there has been a paralysis of those branches supplying the sensation to the cornea, there may be a small amount of pain, and the intolerance of light will be correspondingly small, so that the patient will complain mostly of the obscuration of vision due to the hazy cornea. But in acute keratitis, with either circumscribed or phlyctenular ulcers, where suppuration has extended through the epithelium, and exposed the minute nerves, the pain will be intense, and the photophobia so great that the patient will bury his head beneath pillows, or seek a dark room, and shun all light. There will also be considerable ciliary, and some superorbital neuralgia.

Some of the causes of inflammation and ulceration of the cornea, are defective nutrition or want of assimilation, an impoverished system, cold, or exposure to rough winds, or the contact with the cornea of strong chemicals which are used in the treatment of diseases of the lids, the injudicious use of strong solutions of silver or copper, the contact of foreign bodies with the cornea, or the loss of nutrition to the structure by the paralysis of the nerves in this region.

The danger arising from this affection, are loss of substance, either sufficient to produce perforation of the cornea and prolapse of the iris, or sufficient thinning of the cornea, to produce a bulging outward, from the intraocular pressure, thereby causing a permanent conical cornea, or anterior staphyloma; or a remaining cicatrix, after the healing of the ulcer, which leaves an opacity. When the latter happens to be situated in front of the pupil, the vision is impaired unless a portion of the iris is removed, and the pupil enlarged sufficient to allow the rays of light to enter through the clear cornea.

Where ulceration has been deep and the internal layers of the cornea have been involved, the pus will either burrow in between the different layers of the cornea—a condition called interstitial keratitis—or some of the pus will get into the anterior chamber and be found to gravitate to the bottom, forming a hypopion. In order to tell whether the pus lies between the layers of the cornea, or in the anterior chamber, we have but to remember that in the latter condition, the pus will have a level, and straight upper margin, and that the line of level will change according as the patient's head is kept still or tilted to one side; while if the pus be between the different layers of the cornea the line of level, if there be any, will not change with the movements of the head.

With regard to the treatment of keratitis, it is first necessary to remember, that in nearly every case the patient's general health will be found to be either so low as to constitute the direct cause of the disease, or it will be so much below par that all efforts in treatment will prove of no avail until the general health is looked after.—A tonic course, then, is nearly always advisable, and it is specially indicated if the patient himself feels weak and run down. Preparations of iron and quinine are very valuable adjuvants to the local treatment, and quinine alone or in combination with morphia will be found very beneficial, in the relief of the photophobia, and the neuralgia, which usually follows the course of the branches of the fifth nerve, on the side affected; solutions of atropine, qr. ij. to iv., to aquæ ℥i. should be dropped into the eye every one, two or three hours, to keep the pupils well dilated, and lessen the secretion of the aqueous. The eye should be well protected from light; to accomplish this end a shade or pad should be worn over the eye, or the room darkened during the day. In addition to the local use of atropine, when the ulcer has passed the first stage, and the spreading from the edges has ceased, it is advisable to dust on dry calomel in very fine powder. This gently stimulates the tissues and causes a gradual narrowing from day to day, of the abraded surface, as well as a filling up from the bottom. I have found that in young persons the opacity which is nearly always left after ulcerations, may frequently be prevented in a great measure, and old opacities in children, when not too extensive, may be removed, by the use of solution of soda sulphas ʒj.—aquæ ℥j. It requires sometimes months to effect any marked change in an old opacity, and if the patient is of adult years the opacity remains, but in children, great improvement seems to follow the above course of treatment. With regard to the treatment of accumulations of pus, either between the layers of the cornea, or in the anterior chamber, one end is to be sought, viz: to get rid of the pus by an evacuation. If the accumulation is beneath the first layer of the cornea, and crowds it out, an oblique incision should be made in such a manner as to secure the evacuation of the pus and at the same time not penetrate with the knife the anterior chamber; but for hypopion accumulation of pus in the anterior chamber a paracentesis of the cornea should be made, and allow all the aqueous to flow out together with the pus. The knife should be thrust directly through the cornea, in the seat of the ulcer, rather than at the bottom of the cornea, for the pus will mount up if the paracentesis be made above the center of the cornea, and flow out with the aqueous. Great care should be taken not to wound the iris which will come forward against the cornea while the aqueous runs out. Remarkable effects sometimes follow a paracentesis. I have seen a violent inflammation of the cornea rapidly subside after the pus and aqueous were evacuated daily for two or three days. Sometimes eight or ten evacuations are made before the chamber is finally allowed to remain filled. Whenever there is pus in the anterior chamber, one should not hesitate to let it out by a puncture. When ulceration of the cornea is complicated with some disease of the lids, it is not advisable to attempt to treat both at once. Even mild astringents are to be avoided until the acute stage of ulceration is past, for they always aggravate the symptoms, and cause an increase of the sloughing surface.

*Iritis.*—Iritis, like keratitis, is usually a very painful affection of the eye. It may exist alone or in connection with the disease just described. There are several forms of iritis, which for the most part take their names from the circumstances which combine to cause the disease. Some authors give only one or two varieties, holding that nearly all forms are the result of constitutional infection from syphilis; while others distinguish several forms which are all more or less identical. Four forms are sufficiently well marked to justify distinction: simple or idiopathic iritis, suppurative or parenchymatous iritis, traumatic iritis and syphilitic iritis. The first form is the most mild in its attack and the shortest in its duration, usually being characterized by slight injection of the globe and some pain and dread of light, which become more marked upon the application of a solution of atropine to the eye, when it will be observed that lymph has been thrown out and two or three adhesions have been formed between the iris and the lens. The pupil will therefore dilate very irregularly. In suppurative iritis the parenchymatous substance of the iris will be attacked, and destructive inflammation will cause masses of pus and lymph to be thrown down, forming the accumulation of pus in the anterior chamber known as hypopion. The traumatic kind may follow cataract operations or wounds of the iris by foreign bodies. Its course and termination is very similar to some of the other forms, and need not here be fully discussed. It may lead to suppuration and destruction, or may form posterior attachments to the lens, by the exudation of lymph around the pupillary margin.—Among all the causes which produce the disease, perhaps the most frequent is syphilis. Some authors hold that three-fourths of the cases are of this kind, and it is certainly safe to say, that they constitute by far the majority. The symptoms of iritis are of two kinds: subjective, or those which the patient will observe, and objective, those which the surgeon will see. Among the first will be found the most constant and frequently the first observed, dimness of vision, which is caused by the cloudiness or turbidity of the aqueous. Pain is a very frequent though not invariable symptom. It is nearly always termed by the patient neuralgic. Sometimes it is periodic in recurrence, and called "*sun pain.*" When of this form, quinine is a certain and unfailing specific. Pain, however, is not always present; it may amount only to an uneasy feeling, or itching sensation, or may be most severe and unenduring. Intolerance of light and increased lachrymation are frequently present.—Among the objective symptoms are sluggishness or immobility of the pupil, loss of brilliancy in the iris, as well as loss of striation, together with discoloration; the iris presenting a dull hazy or velvety appearance, and not responding to varying degrees of light. The aqueous is almost always turbid, but the cornea is usually clear. The scleral ring or zone is injected, presenting usually a pinkish appearance. In iritis due to syphilis there will be in addition to these symptons, the characteristic gummy tumors of syphilis developed upon the iris usually not far from its pupillary margin. These tumors are developed from the the fibrous stroma of the, iris and are composed of nodular collections of the products of inflammation, containing piquent granules and blood vessels. They are usually known by the name of condylomatous growths.

After iritis has progressed for several days it will usually be found that small beads of plastic lymph have been exuded all around the border of the pupil. These individual exudations increase in size and coalesce, until they have formed around the entire free margin of the pupil a firm union between the posterior portion of the iris and the capsule of the lens. This union is usually first detected upon the use of atropine in the eye, when the pupil will be found to dilate very irregularly if the union be incomplete, and not at all if there be a complete circular adhesion. This condition is called circular, or annular synechia, or exclusion of the pupil. This is not to be confounded with a condition in which the exudation extends across the pupil and forms a block or plug of lymph in the pupil This is called occlusion, and when complete, always prevents the light from entering the eye, and consequently the sight is proportionately deteriorated. In exclusion, although there may be no motion whatever of the iris on account of its attachments, yet there may still be left a small clear space in the pupil through which light may pass, suffieient to allow of distinct vision ; while this is not the case in occlusion.

Inflammation of the ciliary body often exists with iritis, constituting a complication termed irido-cyklitis. It is brought about by the inflammation progressing till it reaches the peripheral portions of the iris, where the fibrous stroma are in contact with and continuous with the ciliary body. When this form is present, the vascular zone or ring will be found very much injected.

In the treatment of this disease several ends are to be sought; first, the removal of the cause when it is found to be specific in its nature; second, to keep removed all sources of injury, which may keep up inflammation ; third, to diminish the proliferation of tissue cells which go to build up the excrescences which have been described as frequently developing upon the iris, and fourth to prevent the neoplastic formations, which have been described as synechiæ. In handling a syphilitic iritis the specific treatment for that disease will be found to produce very marked beneficial changes in the condition of the iris. The hypodermic use of the bichloride of mercury is advisable ; to be used up to the point of merely showing its effects upon the gums, when the iodide of potash should be used. It is held by the very best authorities, that marked salivation is by no means necessary. Mercury in almost any form, alternated with or given at the same time with iodine, has long been acknowledged to be among the best therapeutic means for the arrest of iritis.—In order to remove any cause which may keep up inflammation, we should see that there be no foreign body upon the iris. Oftentimes this may cause the disease and be overlooked. The corneal wound may be quite small and overlooked, and a fine piece of steel or iron may be lodged upon the iris, causing all the trouble. Hence one may not be too careful in making close inspection when the case is first seen. The third indication demands a local antiphlogistic measure, which may be accomplished in two ways; first, by the active use of atropine, which if the pupil dilates freely and fully, causes the blood to be driven out of the iris, thereby preventing excessive nutrition and hyperæmia which are necessary to the excessive proliferation of tissue cells; second, by the application of leeches to the inner canthus of the eye or to the temple, or by the use of cups to the temple. The object to be sought is only to relieve the intense injection and intraocular pressure of the globe. The fourth indication is best obtained by atropine. When the pupil is widely dilated, the edges of the iris, at the pupiliary margin, float in the aqueous and do not touch the capsule, hence adhesion cannot take place. Oftentimes by the use of the mydriatic, those partial adhesions which have already taken place, will give way, and if not, the irregular dilation of the pupil prevents their farther formation, thus obviating the complete posterior synechia. Astringent collyria are to be avoided, because they are unnecessary. Their presence in the eye does no good, but rather causes increased irritation and consequent hyperæmia of the globe. Hence solutions of silver, zinc, and lead are to be discarded and the eye kept quiet on instillations of atropine, together with the internal use of opium for the severe pain, and the internal use of specifics with the view of removing the cause of the disease. The usual results of a chronic iritis are, exclusion or occlusion of the pupil. When the acute symptoms have passed away, an artificial pupil may be made by the usual method described in the books as iridectomy.—*Indiana Journal of Medicine.*

